# Supplementary material for: Endangered predators and endangered prey: Seasonal diet of Southern Resident killer whales
Source: PLoS One. 2021 Mar 3;16(3):e0247031. doi: 10.1371/journal.pone.0247031 (PMC7928517; doi:10.1371/journal.pone.0247031)
Supplement: S1 Table — Sample number, whale pod identification from genotyping, and proportional contribution of prey species by region of the study area where samples were collected. (DOCX) [file pone.0247031.s001.docx]

**S1 Table. Southern Resident killer whale fecal sample collection information.** Sample number, whale pod identification from genotyping, and proportional contribution of prey species by region of the study area where samples were collected.

| Description of column headings: |  |
| --- | --- |
| Sample ID: | Internal field sample reference number |
| SRKW pod ID | whale pod identification from genotyping |
| Sample composition type | Individual, Extra replicates of individuals, PCR replicates of individuals, or pooled |
| N | Number of samples included each sample run |

a. Puget Sound

Proportional contribution to diet by prey species

b. JdF/SJI

Proportional contribution to diet by prey species

c. Outer coast waters

Proportional contribution to diet by prey species
