# Supplementary material for: Endangered predators and endangered prey: Seasonal diet of Southern Resident killer whales
Source: PLoS One. 2021 Mar 3;16(3):e0247031. doi: 10.1371/journal.pone.0247031 (PMC7928517; doi:10.1371/journal.pone.0247031)
Supplement: S2 Table — Genbank accession number and species name for the 403 fish species found on the U.S. west coast that were used in the analyses of prey in Southern Resident killer whale fecal samples. (DOCX) [file pone.0247031.s002.docx]

**S2 Table. Fish Voucher Specimen Names.** Genbank accession number and species name for the 403 fish species found on the U.S. west coast that were used in the analyses of prey in Southern Resident killer whale fecal samples.

| Genbank accession | Genus | Species |
| --- | --- | --- |
| KJ010762 | Lampetra | tridentata |
| EU685093 | Orcinus | orca |
| EF119242 | Porichthys | notatus |
| EF119243 | Porichthys | notatus |
| EF119244 | Hydrolagus | colliei |
| EF119279 | Hydrolagus | colliei |
| KJ010603 | Bathyraja | trachura |
| KJ010750 | Bathyraja | trachura |
| KJ010702 | Bathyraja | interrupta |
| EF119331 | Bathyraja | interrupta |
| EF458339 | Bathyraja | interrupta |
| KJ010701 | Bathyraja | interrupta |
| KJ010669 | Raja | inornata |
| KJ010670 | Raja | inornata |
| EF119329 | Raja | binoculata |
| EF119333 | Raja | binoculata |
| EF119277 | Raja | rhina |
| EF119278 | Raja | rhina |
| EF119259 | Raja | rhina |
| EF119240 | Squalus | acanthias |
| EF119334 | Squalus | acanthias |
| EF119335 | Squalus | acanthias |
| KJ010677 | Mustelus | henlei |
| KJ010668 | Parmaturus | xaniurus |
| EF119328 | Apristurus | brunneus |
| KJ010587 | Apristurus | brunneus |
| KJ010588 | Apristurus | brunneus |
| KJ010681 | Chauliodus | macouni |
| KJ010637 | Syngnathus | leptorhynchus |
| KJ010638 | Syngnathus | leptorhynchus |
| EF458389 | Engraulis | mordax |
| EF458390 | Engraulis | mordax |
| EF458391 | Engraulis | mordax |
| KJ010622 | Lestidiops | ringens |
| EF458478 | Benthalbella | dentata |
| KJ010553 | Benthalbella | linguidens |
| KJ010550 | Alepisaurus | ferox |
| KJ010558 | Lestidiops | ringens |
| KJ010667 | Synodus | lucioceps |
| KR779477 | Clupea | pallasii |
| EF119254 | Clupea | pallasii |
| EF458341 | Clupea | pallasii |
| EF458434 | Clupea | pallasii |
| EF458374 | Alosa | sapidissima |
| EF458375 | Alosa | sapidissima |
| EF458392 | Sardinops | sagax |
| EF458393 | Sardinops | sagax |
| KJ010602 | Coryphaenoides | acrolepis |
| KJ010691 | Nezumia | stelgidolepis |
| KJ010551 | Nezumia | liolepis |
| KJ010688 | Nezumia | stelgidolepis |
| KJ010671 | Coryphaenoides | cinereus |
| KJ010678 | Coryphaenoides | cinereus |
| KJ010676 | Coryphaenoides | acrolepis |
| KJ010606 | Albatrossia | pectoralis |
| KJ010607 | Albatrossia | pectoralis |
| KJ010675 | Coryphaenoides | acrolepis |
| KR779490 | Laemonema | longipes |
| KJ010629 | Physiculus | rastrelliger |
| KJ010614 | Melanonus | zugmayeri |
| KJ010749 | Melanonus | zugmayeri |
| EF458337 | Merluccius | productus |
| EF458338 | Merluccius | productus |
| KJ010584 | Gadus | chalcogrammus |
| EF119324 | Gadus | chalcogrammus |
| KJ010660 | Gadus | chalcogrammus |
| EF458431 | Microgadus | proximus |
| EF119286 | Microgadus | proximus |
| EF119248 | Microgadus | proximus |
| EF119287 | Microgadus | proximus |
| KJ010577 | Boreogadus | saida |
| EF458334 | Gadus | macrocephalus |
| EF458462 | Gadus | macrocephalus |
| EF458335 | Gadus | macrocephalus |
| EF458442 | Gadus | macrocephalus |
| KR779486 | Gadus | macrocephalus |
| KJ010724 | Antimora | microlepis |
| KJ010725 | Antimora | microlepis |
| KJ010556 | Halargyreus | johnsonii |
| KR779505 | Halargyreus | johnsonii |
| KJ010663 | Halargyreus | johnsonii |
| KJ010559 | Aristostomias | scintillans |
| EF458479 | Aristostomias | scintillans |
| KJ010570 | Talismania | bifurcata |
| KJ010557 | Sagamichthys | abei |
| KJ010757 | Alepocephalus | tenebrosus |
| EF458405 | Catostomus | macrocheilus |
| EF458406 | Ptychocheilus | oregonensis |
| EF458407 | Ptychocheilus | oregonensis |
| EF458408 | Mylocheilus | caurinus |
| EF458411 | Mylocheilus | caurinus |
| KJ010630 | Chilara | taylori |
| EF458347 | Spirinchus | starksi |
| EF458387 | Hypomesus | pretiosus |
| EF458388 | Hypomesus | pretiosus |
| KJ010581 | Osmerus | mordax |
| KJ010728 | Osmeridae | sp. |
| KR779484 | Allosmerus | elongatus |
| EF458343 | Thaleichthys | pacificus |
| EF458344 | Thaleichthys | pacificus |
| KJ010579 | Thaleichthys | pacificus |
| KJ010732 | Thaleichthys | pacificus |
| KJ010687 | Idiacanthus | antrostomus |
| KR779501 | Idiacanthus | antrostomus |
| KJ010552 | Rhadinesthes | decimus |
| KJ010617 | Stomias | atriventer |
| KJ010751 | Stomias | atriventer |
| EF119299 | Citharichthys | sordidus |
| EF458350 | Citharichthys | stigmaeus |
| EF458373 | Citharichthys | stigmaeus |
| EF458413 | Fundulus | diaphanus |
| EF455489 | Oncorhynchus | gorbuscha |
| KU170134 | Oncorhynchus | kisutch |
| KJ010737 | Oncorhynchus | kisutch |
| KU170132 | Oncorhynchus | kisutch |
| GU018123 | Oncorhynchus | mykiss |
| KR476952 | Oncorhynchus | mykiss |
| KU170133 | Oncorhynchus | kisutch |
| KU170140 | Oncorhynchus | mykiss |
| KJ010736 | Oncorhynchus | clarkii |
| KU170137 | Oncorhynchus | keta |
| KU170138 | Oncorhynchus | keta |
| KU170139 | Oncorhynchus | mykiss |
| KU170128 | Oncorhynchus | tshawytscha |
| KU170129 | Oncorhynchus | tshawytscha |
| KU170130 | Oncorhynchus | tshawytscha |
| KU170131 | Oncorhynchus | tshawytscha |
| KU170135 | Oncorhynchus | nerka |
| KU170136 | Oncorhynchus | nerka |
| KJ010561 | Scopelengys | tristis |
| KJ010649 | Diaphus | theta |
| KJ010641 | Nannobrachium | regale |
| KJ010659 | Nannobrachium | regale |
| KR779503 | Stenobrachius | leucopsarus |
| KR779504 | Macropinna | microstoma |
| KJ010710 | Leuroglossus | schmidti |
| EF458484 | Lipolagus | ochotensis |
| KJ010747 | Poromitra | crassiceps |
| KJ010652 | Poromitra | crassiceps |
| KJ010628 | Melamphaes | lugubris |
| KJ010627 | Melamphaes | lugubris |
| EF458476 | Allocyttus | folletti |
| KJ010616 | Anoplogaster | cornuta |
| KJ010643 | Barbourisia | rufa |
| KJ010562 | Rondeletia | loricata |
| KJ010567 | Rondeletia | loricata |
| KJ010672 | Hippoglossina | stomata |
| KJ010615 | Hippoglossina | stomata |
| EF119320 | Atheresthes | stomias |
| KR779483 | Atheresthes | stomias |
| KJ010604 | Embassichthys | bathybius |
| KJ010683 | Embassichthys | bathybius |
| EF458432 | Eopsetta | jordani |
| KJ010572 | Limanda | aspera |
| KJ010580 | Limanda | aspera |
| KJ010578 | Limanda | sakhalinensis |
| EF119261 | Hippoglossoides | elassodon |
| KJ010589 | Microstomus | pacificus |
| EF119300 | Glyptocephalus | zachirus |
| EF119301 | Glyptocephalus | zachirus |
| KJ010583 | Limanda | proboscidea |
| EF119289 | Parophrys | vetulus |
| EF119288 | Parophrys | vetulus |
| EF458352 | Isopsetta | isolepis |
| EF119330 | Isopsetta | isolepis |
| EF119241 | Psettichthys | melanostictus |
| EF458443 | Lepidopsetta | polyxystra |
| EF119260 | Psettichthys | melanostictus |
| EF458340 | Platichthys | stellatus |
| EF458356 | Hippoglossus | stenolepis |
| EF119275 | Lyopsetta | exilis |
| KJ010666 | Pleuronichthys | verticalis |
| EF119270 | Pleuronichthys | coenosus |
| EF458438 | Pleuronichthys | coenosus |
| EF458382 | Pleuronichthys | decurrens |
| KR779487 | Pleuronichthys | decurrens |
| KJ010599 | Gigantactis | vanhoeffeni |
| KJ010554 | Chaenophryne | longiceps |
| KJ010753 | Oneirodes | sp. |
| KJ010566 | Oneirodes | bulbosus |
| KJ010761 | Lepidogobius | lepidus |
| KJ010756 | Paralabrax | nebulifer |
| EF458384 | Cololabis | saira |
| EF458385 | Cololabis | saira |
| KJ010661 | Lamprogrammus | niger |
| EF458402 | Atherinops | affinis |
| EF458394 | Scomber | japonicus |
| KJ010717 | Liparidae | sp. |
| EF119256 | Cymatogaster | aggregata |
| KR779499 | Brachyistius | frenatus |
| EF119255 | Rhacochilus | vacca |
| EF119283 | Rhacochilus | vacca |
| KJ010620 | Zalembius | rosaceus |
| EF119265 | Embiotoca | lateralis |
| EF458475 | Brama | japonica |
| EF458420 | Trachurus | symmetricus |
| KR779498 | Brosmophycis | marginata |
| KJ010623 | Cataetyx | rubrirostris |
| KJ010742 | Cataetyx | rubrirostris |
| EF458452 | Icichthys | lockingtoni |
| KJ010625 | Genyonemus | lineatus |
| KJ010626 | Genyonemus | lineatus |
| KJ010673 | Genyonemus | lineatus |
| KJ010741 | Elassodiscus | caudatus |
| KJ010745 | Elassodiscus | caudatus |
| KJ010655 | Sebastolobus | altivelis |
| KJ010656 | Sebastolobus | altivelis |
| KJ010760 | Sebastolobus | altivelis |
| EF458441 | Sebastolobus | alascanus |
| EF458351 | Sebastolobus | alascanus |
| KJ879093 | Sebastes | alutus |
| EF446602 | Sebastes | levis |
| KJ879044 | Sebastes | levis |
| EF446512 | Sebastes | elongatus |
| EF446511 | Sebastes | elongatus |
| KJ879085 | Sebastes | semicinctus |
| KJ879030 | Sebastes | semicinctus |
| KJ879086 | Sebastes | semicinctus |
| KJ879092 | Sebastes | alutus |
| EF446561 | Sebastes | saxicola |
| EF446502 | Sebastes | saxicola |
| EF446523 | Sebastes | saxicola |
| EF446519 | Sebastes | diploproa |
| EF446574 | Sebastes | diploproa |
| KJ879022 | Sebastes | diploproa |
| KJ879039 | Sebastes | aurora |
| EF446579 | Sebastes | aurora |
| EF446583 | Sebastes | atrovirens |
| EF446600 | Sebastes | macdonaldi |
| KJ879141 | Sebastes | melanostomus |
| KJ879127 | Sebastes | ruberrimus |
| KJ879124 | Sebastes | ruberrimus |
| KJ879125 | Sebastes | ruberrimus |
| KJ879175 | Sebastes | diploproa |
| EF446513 | Sebastes | proriger |
| EF446594 | Sebastes | brevispinis |
| EF446589 | Sebastes | zacentrus |
| EF446504 | Sebastes | emphaeus |
| EF446492 | Sebastes | aleutianus |
| EF446501 | Sebastes | proriger |
| KJ010601 | Sebastes | glaucus |
| KJ879152 | Sebastes | miniatus |
| EF446598 | Sebastes | pinniger |
| KJ879183 | Sebastes | goodei |
| EF446548 | Sebastes | alutus |
| KJ879128 | Sebastes | alutus |
| EF446569 | Sebastes | dallii |
| EF446572 | Sebastes | jordani |
| EF446578 | Sebastes | ensifer |
| KJ879100 | Sebastes | chlorostictus |
| EF446550 | Sebastes | constellatus |
| KJ879148 | Sebastes | lentiginosus |
| KJ879075 | Sebastes | babcocki |
| EF446515 | Sebastes | flavidus |
| EF446553 | Sebastes | ensifer |
| KJ879142 | Sebastes | ensifer |
| KJ879165 | Sebastes | ensifer |
| KJ879160 | Sebastes | rosaceus |
| KJ879034 | Sebastes | maliger |
| EF446567 | Sebastes | serriceps |
| KJ879120 | Sebastes | maliger |
| KJ879035 | Sebastes | auriculatus |
| EF446588 | Sebastes | chrysomelas |
| EF446599 | Sebastes | maliger |
| KJ879052 | Sebastes | entomelas |
| KJ879023 | Sebastes | paucispinis |
| KJ879024 | Sebastes | crameri |
| KJ879184 | Sebastes | crameri |
| KJ010716 | Aulorhynchus | flavidus |
| KJ010573 | Ammodytes | hexapterus |
| KJ010612 | Paraliparis | cephalus |
| KJ010610 | Paraliparis | rosaceus |
| KJ010721 | Liparidae | sp. |
| KJ010658 | Careproctus | sp. |
| KJ010564 | Careproctus | melanurus |
| KJ010555 | Careproctus | cypselurus |
| KJ010653 | Careproctus | sp. |
| KR779500 | Careproctus | cypselurus |
| EF458486 | Careproctus | cypselurus |
| KJ010569 | Careproctus | gilberti |
| KJ010624 | Paraliparis | dactylosus |
| KJ010744 | Paraliparis | dactylosus |
| EF458412 | Gasterosteus | aculeatus |
| EF458451 | Liparis | fucensis |
| EF458437 | Anoplopoma | fimbria |
| EF458482 | Anoplopoma | fimbria |
| KR779511 | Liparis | rutteri |
| EF458440 | Liparis | pulchellus |
| EF458398 | Liparis | florae |
| KJ010700 | Liparis | sp. |
| EF458363 | Liparis | dennyi |
| KJ010697 | Liparis | sp. |
| EF458450 | Liparis | dennyi |
| KJ010698 | Liparis | sp. |
| KJ010699 | Liparis | sp. |
| EF119317 | Nautichthys | oculofasciatus |
| KR779478 | Nautichthys | oculofasciatus |
| EF119313 | Rhamphocottus | richardsoni |
| EF458433 | Rhamphocottus | richardsoni |
| EF458379 | Eumicrotremus | orbis |
| EF458378 | Eumicrotremus | orbis |
| KR779489 | Oxylebius | pictus |
| KJ010619 | Zaniolepis | frenata |
| KJ010733 | Zaniolepis | latipinnis |
| KJ010596 | Pholis | laeta |
| KJ010597 | Pholis | laeta |
| KJ010712 | Pholis | laeta |
| KJ010713 | Pholis | laeta |
| KJ010575 | Bathymaster | signatus |
| EF458355 | Cryptacanthodes | giganteus |
| EF458480 | Zaprora | silenus |
| KJ010648 | Poroclinus | rothrocki |
| EF119322 | Lumpenus | sagitta |
| EF119323 | Lumpenus | sagitta |
| EF458401 | Anarrhichthys | ocellatus |
| EF458460 | Chirolophis | decoratus |
| EF458436 | Ronquilus | jordani |
| KJ010719 | Melanostigma | pammelas |
| KJ010718 | Melanostigma | pammelas |
| KJ010563 | Lycenchelys | crotalinus |
| KJ010568 | Lycodes | cortezianus |
| EF119257 | Lycodes | pacificus |
| KJ010689 | Lycodes | diapterus |
| KJ010586 | Lycodapus | mandibularis |
| EF458485 | Lycodapus | fierasfer |
| EF458477 | Lycodapus | fierasfer |
| EF458345 | Lycodes | brevipes |
| KJ010608 | Bothrocara | brunneum |
| EF458428 | Lycodes | palearis |
| EF458446 | Lycodes | palearis |
| KJ010707 | Anoplagonus | inermis |
| KJ010706 | Anoplagonus | inermis |
| EF458403 | Trichodon | trichodon |
| KJ010650 | Trichodon | trichodon |
| EF458472 | Artedius | harringtoni |
| EF458473 | Artedius | harringtoni |
| KJ010592 | Artedius | fenestralis |
| EF119306 | Chitonotus | pugetensis |
| EF119307 | Chitonotus | pugetensis |
| KJ010726 | Psychrolutes | paradoxus |
| EF119250 | Leptocottus | armatus |
| KJ010738 | Cottus | confusus |
| EF458399 | Cottus | asper |
| EF458424 | Icelinus | filamentosus |
| EF458457 | Icelinus | filamentosus |
| KJ010611 | Icelinus | fimbriatus |
| EF458368 | Icelinus | borealis |
| EF458346 | Icelinus | borealis |
| EF458463 | Malacocottus | kincaidi |
| EF458464 | Malacocottus | kincaidi |
| EF119247 | Myoxocephalus | polyacanthocephalus |
| EF119314 | Myoxocephalus | polyacanthocephalus |
| EF119271 | Myoxocephalus | polyacanthocephalus |
| KJ010752 | Enophrys | taurina |
| EF119332 | Enophrys | bison |
| EF119318 | Dasycottus | setiger |
| EF458342 | Triglops | macellus |
| EF458376 | Triglops | macellus |
| KJ010696 | Radulinus | boleoides |
| EF458429 | Radulinus | asprellus |
| KJ010730 | Radulinus | asprellus |
| KR779494 | Triglops | pingelii |
| EF458365 | Triglops | pingelii |
| KJ010686 | Triglops | pingelii |
| EF458349 | Hexagrammos | decagrammus |
| KJ010600 | Pleurogrammus | monopterygius |
| KR779488 | Pleurogrammus | monopterygius |
| EF119319 | Hexagrammos | stelleri |
| KR779492 | Hexagrammos | stelleri |
| EF458353 | Ophiodon | elongatus |
| KJ010705 | Scorpaenichthys | marmoratus |
| KJ010636 | Bothragonus | swanii |
| KR779510 | Agonopsis | vulsa |
| EF458381 | Agonopsis | vulsa |
| EF458380 | Agonopsis | vulsa |
| EF119269 | Agonopsis | vulsa |
| KJ010694 | Pallasina | barbata |
| EF458357 | Pallasina | barbata |
| KJ010695 | Pallasina | barbata |
| KJ010714 | Blepsias | cirrhosus |
| KJ010613 | Chesnonia | verrucosa |
| KJ010692 | Chesnonia | verrucosa |
| EF119253 | Podothecus | accipenserinus |
| EF119264 | Podothecus | accipenserinus |
| EF458466 | Bathyagonus | nigripinnis |
| KJ010631 | Xeneretmus | leiops |
| KJ010594 | Xeneretmus | latifrons |
| KJ010565 | Bathyagonus | pentacanthus |
| EF458422 | Bathyagonus | pentacanthus |
| KR779496 | Hypsagonus | quadricornis |
| KR779502 | Alosa | sapidissima |
| EF458358 | Hemilepidotus | hemilepidotus |
| EF458449 | Hemilepidotus | spinosus |
| KR779493 | Hemilepidotus | spinosus |
| EF458370 | Hemilepidotus | spinosus |
| EF119294 | Peprilus | simillimus |
| EF458430 | Peprilus | simillimus |
| EF119295 | Peprilus | simillimus |
| KJ010571 | Aphanopus | arigato |
| KJ010618 | Chiasmodon | niger |
| KJ010722 | Chiasmodon | niger |
| KJ010560 | Kali | kerberti |
| KJ010646 | Tetragonurus | cuvieri |
