# Supplementary material for: Endangered predators and endangered prey: Seasonal diet of Southern Resident killer whales
Source: PLoS One. 2021 Mar 3;16(3):e0247031. doi: 10.1371/journal.pone.0247031 (PMC7928517; doi:10.1371/journal.pone.0247031)
Supplement: S3 Table — Summary of sampling effort and sample type recovered from Southern resident killer whales during October to May 2004 to 2017 in Puget Sound (PS), and Juan de Fuca Strait/San Juan Islands (JdF/SJI), Northern Georgia Strait (NGS), and the outer coast waters of Washington, Oregon, and California (CW), study areas. (DOCX) [file pone.0247031.s003.docx]

**S3 Table. Southern Resident killer whale diet effort and sample summary.** Annual summary of sampling effort and sample type recovered from Southern Resident killer whales during October to May 2004 to 2017 in Puget Sound (PS), and Juan de Fuca Strait/San Juan Islands (JdF/SJI), Northern Georgia Strait (NGS), and the outer coast waters of Washington, Oregon, and California (CW), study areas.

| Year  Fall-Winter/  spring | Months samples collected | | | | Days of Effort | | | | Number of prey remains samples (scales and/or tissues) | | | | Number of fecal samples | | | | |
| --- | --- | --- | --- | --- | --- | --- | --- | --- | --- | --- | --- | --- | --- | --- | --- | --- | --- |
|  | PS | JdF/  SJI | NGS | CW | PS | JdF/SJI | NGS | CW | PS | JdF/  SJI | NGS | CW | PS | JdF/SJI | NGS | CW |  |
| 2003-4 |  |  |  | Mar |  |  |  | 1 |  |  |  | 0 |  |  |  | 0 |  |
| 2004-5 |  |  |  |  |  |  |  |  |  |  |  |  |  |  |  |  |  |
| 2005-6 | Oct, Nov, Dec | Mar |  | Mar | 8 | 1 |  | 1 | 10 ^a^ | 0 |  | 0 | 0 | 0 |  | 0 |  |
| 2006-7 | Oct, Nov, Dec |  |  |  | 6 |  |  |  | 7 |  |  |  | 0 |  |  |  |  |
| 2007-8 | Nov, Dec, Jan | Mar |  | Oct | 20 | 1 |  | 1 | 22^b,c^ | 0 |  | 0 | 14 | 0 |  | 0 |  |
| 2008-9 | Dec, Feb, Mar |  |  | Mar | 8 |  |  | 2 | 9 | 1 |  | 2 | 10 | 0 |  | 0 |  |
| 2009-10 | Oct, Dec, Jan | Oct |  |  | 11 | 1 |  |  | 12^d^ | 0 |  |  | 0 | 0 |  |  |  |
| 2010-11 | Oct, Nov, Feb, May | Oct |  |  | 11 | 1 |  |  | 16^a,b,e^ | 0 |  |  | 6 | 5 |  |  |  |
| 2011-12 | Oct, Dec, Jan | Oct |  | Apr, Jun | 5 | 2 |  | 2 |  | 1 |  | 0 | 5 | 0 |  | 0 |  |
| 2012-13 | Oct, Nov, Dec, Jan | Jan |  | Jan, Mar | 9 | 1 |  | 11 | 8 | 1 |  | 21 | 0 | 0 |  | 18 |  |
| 2013-14 | Oct, Nov, Dec, Jan, Apr |  |  |  | 12 |  |  |  | 5 |  |  | 1 | 7 |  |  |  |  |
| 2014-15 | Oct, Nov, Dec, Jan |  | Feb | Feb, Mar, Apr, May | 7 |  | 3 | 15 | 1 |  | 2 | 30^a^ | 6 |  | 0 | 9 |  |
| 2015-16 | Nov, Dec, Jan | Mar |  | Feb | 8 | 1 |  | 3 | 0 | 0 |  | 0 | 0 | 0 |  | 1 |  |
| 2016-17 | Oct, Nov | Mar |  |  | 3 | 1 |  |  | 3 | 0 |  | 0 | 0 | 0 |  |  |  |
| Total samples |  |  |  |  | 108 | 9 | 3 | 36 | 93 | 3 | 2 | 54 | 48 | 5 | 0 | 28 |  |

a - Samples contained scales/tissues for two different fish species. b -Samples contained two different Chinook stocks. c – Two sample’s IDs failed. d – One sample’s ID failed. e – One sample was regurgitation which included the scales of two different fish species.
