# Supplementary material for: Endangered predators and endangered prey: Seasonal diet of Southern Resident killer whales
Source: PLoS One. 2021 Mar 3;16(3):e0247031. doi: 10.1371/journal.pone.0247031 (PMC7928517; doi:10.1371/journal.pone.0247031)
Supplement: S4 Table — (DOCX) [file pone.0247031.s004.docx]

**S4 Table. Observed and expected species composition of experimental controls for Southern Resident killer whale prey species.**

| Common name | Big skate | Pacific herring | Pacific sanddab | Pink salmon | Coho salmon | Steelhead | Chum salmon | Chinook salmon | Sockeye salmon | Arrowtooth flounder | Dover sole | English sole | Pacific sand sole | Pacific halibut | Lingcod | other |
| --- | --- | --- | --- | --- | --- | --- | --- | --- | --- | --- | --- | --- | --- | --- | --- | --- |
| Scientific name | Raja binoculata | Clupea pallasii | Citharichthys sordidus | Oncorhynchus gorbuscha | Oncorhynchus kisutch | Oncorhynchus mykiss | Oncorhynchus keta | Oncorhynchus tshawytscha | Oncorhynchus nerka | Atheresthes stomias | Microstomus pacificus | Parophrys vetulus | Psettichthys melanostictus | Hippoglossus stenolepis | Ophiodon elongatus | other |
| Sample ID |  |  |  |  |  |  |  |  |  |  |  |  |  |  |  |  |
| Control-1 | 0.0002 | 0.0000 | 0.0000 | 0.0000 | 0.2417 | 0.0011 | 0.0008 | 0.2340 | 0.0007 | 0.0000 | 0.0061 | 0.0000 | 0.0004 | 0.2961 | 0.2091 | 0.0097 |
| Control-1-2 | 0.0001 | 0.0000 | 0.0000 | 0.0000 | 0.2258 | 0.0009 | 0.0003 | 0.2284 | 0.0011 | 0.0000 | 0.0073 | 0.0000 | 0.0005 | 0.2931 | 0.2317 | 0.0108 |
| Control-1-3 | 0.0001 | 0.0000 | 0.0000 | 0.0000 | 0.2298 | 0.0009 | 0.0004 | 0.2280 | 0.0010 | 0.0000 | 0.0071 | 0.0001 | 0.0006 | 0.2862 | 0.2356 | 0.0103 |
| Control 1 - mean | 0.0001 | 0.0000 | 0.0000 | 0.0000 | 0.2324 | 0.0010 | 0.0005 | 0.2301 | 0.0009 | 0.0000 | 0.0068 | 0.0000 | 0.0005 | 0.2918 | 0.2255 | 0.0103 |
| Control 1 - standard error | 0.0000 | 0.0000 | 0.0000 | 0.0000 | 0.0048 | 0.0000 | 0.0001 | 0.0019 | 0.0001 | 0.0000 | 0.0004 | 0.0000 | 0.0001 | 0.0030 | 0.0083 | 0.0003 |
| Expected - control 1 | 0.0000 | 0.0000 | 0.0000 | 0.0000 | 0.2500 | 0.0000 | 0.0000 | 0.2500 | 0.0000 | 0.0000 | 0.0000 | 0.0000 | 0.0000 | 0.2500 | 0.2500 | 0.0000 |
| Mean - expected | 0.0001 | 0.0000 | 0.0000 | 0.0000 | -0.0176 | 0.0010 | 0.0005 | -0.0199 | 0.0009 | 0.0000 | 0.0068 | 0.0000 | 0.0005 | 0.0418 | -0.0245 | 0.0103 |
| Control-2 | 0.0001 | 0.0599 | 0.0000 | 0.0000 | 0.0025 | 0.0006 | 0.0005 | 0.1169 | 0.0007 | 0.0000 | 0.0124 | 0.0001 | 0.0006 | 0.4014 | 0.3954 | 0.0089 |
| Control-2-2 | 0.0005 | 0.0592 | 0.0000 | 0.0000 | 0.0021 | 0.0005 | 0.0004 | 0.1087 | 0.0005 | 0.0000 | 0.0106 | 0.0001 | 0.0005 | 0.4063 | 0.4035 | 0.0072 |
| Control-2-3 | 0.0001 | 0.0532 | 0.0000 | 0.0001 | 0.0021 | 0.0005 | 0.0001 | 0.1019 | 0.0007 | 0.0000 | 0.0117 | 0.0000 | 0.0006 | 0.4186 | 0.4026 | 0.0078 |
| Control 2 - mean | 0.0002 | 0.0574 | 0.0000 | 0.0000 | 0.0022 | 0.0005 | 0.0003 | 0.1092 | 0.0006 | 0.0000 | 0.0116 | 0.0001 | 0.0006 | 0.4088 | 0.4005 | 0.0079 |
| Control 2 standard error | 0.0001 | 0.0021 | 0.0000 | 0.0000 | 0.0002 | 0.0000 | 0.0001 | 0.0043 | 0.0001 | 0.0000 | 0.0005 | 0.0000 | 0.0000 | 0.0051 | 0.0026 | 0.0005 |
| Expected - control 2 | 0.0000 | 0.0500 | 0.0000 | 0.0000 | 0.0000 | 0.0000 | 0.0000 | 0.1500 | 0.0000 | 0.0000 | 0.0000 | 0.0000 | 0.0000 | 0.4000 | 0.4000 | 0.0000 |
| Mean - expected | 0.0002 | 0.0074 | 0.0000 | 0.0000 | 0.0022 | 0.0005 | 0.0003 | -0.0408 | 0.0006 | 0.0000 | 0.0116 | 0.0001 | 0.0006 | 0.0088 | 0.0005 | 0.0079 |
| Control-3 | 0.0003 | 0.0000 | 0.0000 | 0.1135 | 0.2212 | 0.2091 | 0.1083 | 0.2585 | 0.0885 | 0.0000 | 0.0000 | 0.0000 | 0.0000 | 0.0002 | 0.0003 | 0.0000 |
| Control-3-2 | 0.0002 | 0.0000 | 0.0000 | 0.1054 | 0.2249 | 0.2115 | 0.1137 | 0.2580 | 0.0855 | 0.0000 | 0.0000 | 0.0000 | 0.0000 | 0.0003 | 0.0003 | 0.0000 |
| Control-3-3 | 0.0001 | 0.0000 | 0.0000 | 0.1175 | 0.2137 | 0.2136 | 0.1178 | 0.2439 | 0.0928 | 0.0000 | 0.0000 | 0.0000 | 0.0000 | 0.0003 | 0.0003 | 0.0000 |
| Control 2 - mean | 0.0002 | 0.0000 | 0.0000 | 0.1122 | 0.2200 | 0.2114 | 0.1133 | 0.2535 | 0.0889 | 0.0000 | 0.0000 | 0.0000 | 0.0000 | 0.0003 | 0.0003 | 0.0000 |
| Control 2 standard error | 0.0001 | 0.0000 | 0.0000 | 0.0036 | 0.0033 | 0.0013 | 0.0028 | 0.0048 | 0.0021 | 0.0000 | 0.0000 | 0.0000 | 0.0000 | 0.0000 | 0.0000 | 0.0000 |
| Expected - control 3 | 0.0000 | 0.0000 | 0.0000 | 0.1500 | 0.2000 | 0.1500 | 0.1500 | 0.2000 | 0.1500 | 0.0000 | 0.0000 | 0.0000 | 0.0000 | 0.0000 | 0.0000 | 0.0000 |
| Mean - expected | 0.0002 | 0.0000 | 0.0000 | -0.0378 | 0.0200 | 0.0614 | -0.0367 | 0.0535 | -0.0611 | 0.0000 | 0.0000 | 0.0000 | 0.0000 | 0.0003 | 0.0003 | 0.0000 |
