# Supplementary material for: Endangered predators and endangered prey: Seasonal diet of Southern Resident killer whales
Source: PLoS One. 2021 Mar 3;16(3):e0247031. doi: 10.1371/journal.pone.0247031 (PMC7928517; doi:10.1371/journal.pone.0247031)
Supplement: S5 Table — Estimated mean percentage (±SE) of Chinook salmon stock composition of Southern Resident killer whale prey from Puget Sound (PS) and outer coast waters (CW) based on remains from prey capture event samples for February, March, and April. Chinook genetic stock groups are arranged from north to south. Chinook populations from [26], Appendix 1. (DOCX) [file pone.0247031.s005.docx]

**S5 Table. Southern Resident killer whale Chinook prey monthly stock composition.** Estimated mean percentage (±SE) of Chinook salmon stock composition of Southern Resident killer whale prey from Puget Sound (PS) and outer coast waters (CW) based on remains from prey capture event samples for February, March, and April. Chinook genetic stock groups are arranged from north to south. Chinook populations from [26], Appendix 1.

|  | Month | | |
| --- | --- | --- | --- |
| Genetic stock group | February | March | April |
| N | 11 | 17 | 5 |
| Taku R. | 0.0±15.3 |  |  |
| Upper Stikine R. | 10.0±0.5 |  |  |
| So. SEAk - Stikine |  |  |  |
| So. SEAk | 0.0±10.2 | 0.0±9.0 |  |
| Nass R. |  |  |  |
| Upper Skeena R. |  |  |  |
| Lower Skeena R. |  |  |  |
| Cent. BC Coast |  | 0.0±2.4 |  |
| West Vancouver I. |  |  | 0.0±20.2 |
| East Vancouver I. |  |  |  |
| Fraser R. (all) | 0.0 | 5.8 | 19.3 |
| Upper Fraser R. |  |  | 0.0±20.3 |
| Mid Fraser R. |  | 5.8±6.0 | 19.3±18.4 |
| No. Thompson R. |  |  |  |
| So. Thompson | 0.0±9.7 |  |  |
| Lower Thompson R. |  |  |  |
| Lower Fraser R. |  |  |  |
| Puget Sound (all) | 0.0 | 22.9 | 0.0 |
| No. Puget Sound | 0.0±10.0 | 16.9±12.4 | 0.0±10.2 |
| So. Puget Sound |  | 6.0±8.9 |  |
| Juan de Fuca St. |  |  |  |
| Washington coast |  |  |  |
| Columbia R. (all) | 71.8 | 53.7 | 40.6 |
| Snake R. (spring/summer) |  | 5.9±6.0 | 0.0±30.5 |
| Snake R. (fall) | 0.0±10.2 |  |  |
| Upper Col. R. (sum./fall) | 10.1±10.5 | 17.7±9.0 |  |
| Mid/Upper Col R. spring |  |  | 40.6±20.4 |
| Mid. Col. R. Tule | 19.9±15.4 | 4.8±10.2 |  |
| Deschutes R. |  | 0.0±9.7 | 0.0±20.4 |
| Willamette R. | 0.0±4.9 |  | 0.0±20.2 |
| Lower Col. R. spring | 11.6±19.1 | 25.9±9.7 | 0.0±20.4 |
| Lower Col. R. Fall | 30.2±15.2 | 0.0±8.9 | 0.0±16.8 |
| No. Oregon Coast |  |  |  |
| Mid. Oregon Coast | 0.0±9.2 | 0.0±8.7 | 13.6±13.5 |
| Rogue R. | 0.0±1.9 |  |  |
| Klamath R. |  | 5.9±6.0 |  |
| Central Valley Total | 18.3 | 11.7 | 26.6 |
| Central Valley Spring | 18.3±11.0 | 11.7±9.0 | 0.0±20.6 |
| Central Valley Fall | 0.0±15.0 | 0.0±6.0 | 26.6±17.1 |
