# Supplementary material for: Endangered predators and endangered prey: Seasonal diet of Southern Resident killer whales
Source: PLoS One. 2021 Mar 3;16(3):e0247031. doi: 10.1371/journal.pone.0247031 (PMC7928517; doi:10.1371/journal.pone.0247031)
Supplement: Attachment — Submitted filename: PONE-D-20-14399_review.pdf.docx [file pone.0247031.s006.docx]

**Endangered predators and endangered prey: Seasonal diet of SRKW**

**Submitted to PLOS ONE**

**PONE-D-20-14399**

**Comments:**

Thank you for the opportunity to review this interesting and well-written manuscript that documents the seasonal diet of southern resident killer whales. This paper begins with an introduction to the endangered population of resident (fish eating) whales known as the southern resident killer whale (SRKW). A primary threat to recovery is insufficient prey and the authors identify the need for a clear understanding of the seasonal diet as a high conservation priority. Summer diet has been studied previously; this work sets out to characterize diet from October to May and involves collection and analysis of both fecal samples and prey remains.

**Summary of the Manuscript**

**Introduction**

The authors provide an overview of the SRKW population, including the pods, and their known prey preferences. The paper revolves around a key statement, “simultaneously achieving recovery of this predator and its prey while sustaining viable fisheries presents a significant management challenge” and sets out to characterize the whales’ diet during fall, winter, and spring throughout their range from fecal and prey samples.

**Methods**

Prey remains and fecal sample methodology are described elsewhere. Photo IDs of animals present during the sampling period is undertaken to identify population and pod.

Scales from prey remains were analysed to determine species and age (salmonids only); genomic DNA was extracted and species were determined by PCR amplification and sequencing of the mitochondrial genome; sample genotypes at 13 nuclear loci were compared to a coast-wide data set of genotypes representing 42 Chinook salmon populations. Data are expressed as estimates of the percentage of each stock group within the prey samples.

Fecal samples were analysed based on amplification of a mitochondrial 16s gene sequence. Details on individual and pooled sample analysis, validation techniques and control sample analysis are provided. To account for greater variability in winter diet, the authors expanded the 19-species database used for summer diet studies to include 246 species found on the US west coast. Diet patterns are summarized by region and diversity is expressed by number of species present in a sample. Fecal samples were genotyped to identify whale to pod.

**Results**

**Fecal**

A total of 154 samples of predation remains (yielding 158 unique fish) and 81 fecal samples were used in this analysis. Observed mixtures in the known prey composition controls were generally close to expectations and samples displayed little variation in estimates among replicates.

Chinook salmon was the most common species identified (52%) followed by chum salmon and steelhead. Non-salmonids make up 16% of the total prey sequences. Prey species composition varied considerably among fecal samples.

**Predation event samples**

In Puget Sound, the majority of prey remains samples were salmonids with primarily chum salmon in the fall. Coastal samples yielded primarily Chinook. Genetic stock was determined for 20 of 27 Puget Sound Chinook samples and 33 of 44 coastal samples. Fish age based on scale annuli indicate that the majority of chum salmon consumed were 4 years-old. Chinook salmon from Puget Sound were nearly equally distributed among 2, 3, and 4 years-old while coastal waters Chinook were primarily 4 years-old fish.

**Discussion**

**Species Composition of Diet**

The winter data confirm that Chinook represent a major component of diet year round, with differences between species proportions from coastal vs inland waters. Temporal variations in the proportions of other species, as well as differences between samples from coastal to inland waters are explored. Fall diet in inland waters is dominated by chum and coho salmon, while steelhead and lingcod feature more prominently in coastal diets. By April and May, whales return to a mostly Chinook salmon diet.

**Differences in diet results – prey remains and feces**

Prey species in fecal samples are more diverse (three-fold larger number of prey species); prey remains may underestimate the contribution of some species in the diet. The authors provide three potential explanations – 1) fecal represents a number of feeding events rather than a singular predation event; 2) feeding behavior and prey species behavior and distribution will bias presence in sample (e.g., deep water foraging is less likely to result in prey remains at the surface; and 3) prey morphology (e.g., cycloid scales from salmonids may have a different recovery rate than lingcod skin).

**Chinook populations**

The authors report a wide variety of stocks from nearly every major river system from California to SE Alaska are represented – Columbia stocks being particularly prevalent. This is not unexpected given that despite production being a fraction of historic levels, the Columbia still produces more Chinook salmon than any other system on the west coast of North America. These data contrast markedly with Salish Sea summer diet, which consists of primarily Fraser River and Puget Sound stocks. Chinook are generally consumed in relatively close proximity to natal river; however, in non-summer months, some Chinook consumed were quite distant from their natal river.

**Implications – competition from Northern Resident Killer Whales (NRKW)**

The authors note that the NRKW population overlaps in range and consumes some of the same stocks as the SRKW. By comparison, the NRKW has greater stock availability than SRKW. Differences in the size of salmon consumed by each population are noted, with the SRKWs consuming generally younger fish.

**Conservation implications**

The data presented in this paper reinforce that Chinook is of central importance to SRKW but the authors caution that the contribution of, and therefore conservation of, other prey species may be important to recovery. A diverse number of Chinook salmon stocks are consumed in coastal waters with numerous stocks listed as threatened or endangered under the Endangered Species Act. Information on hatchery runs vs wild are provided; the large portion of hatchery Chinook in the winter diet may allow for the potential to manage Chinook salmon stocks to aid in recovery of this endangered population.

**REVIEWER COMMENTS**

Data presented in this paper represent a significant contribution to our understanding of SRKW diet and expand the available information for conservation and management decisions. The authors have collected a spatially diverse library of fecal samples and prey remains throughout the SRKW’s range and over a series of years to amass a detailed overview of SRKW diet and provide narrative on the composition of diet and potential implications for conservation. The methodologies are well described with attention to validation and control samples. The findings are informative with respect to the increased diversity in winter diet, differences between coastal and inland prey base, and the authors provide an interesting discussion on conservation implications of the data. The manuscript is well written, well referenced and a pleasure to read.

The comments on potential SRKW impacts from NRKW competition is intriguing and worthy of further discussion. Readers unfamiliar with the two populations of this fish-eating ecotype would benefit from knowing that the NRKW have maintained an increasing population trajectory of 2% annually and have a four-fold higher population (Towers 2018). In addition, information on body condition from both populations is available; these data are not discussed in this MS, but are relevant in relation to the interesting finding on inter-population differences in stock diversity, differences in age/size of prey and the implications to the energetic cost vs yield of foraging.

Improvement in the quality of the map and addition of the range of the two populations and the locations of some of the primary rivers (Columbia, Fraser, Thompson, Taku, Skeena, Snake, etc) would be beneficial.

The authors present interesting information on the relative proportion of wild vs hatchery stocks and the population’s dependence on these stocks in the spring and fall. In the summer months, prey are dominated by Fraser River stocks, a system comprised of stocks with minimal or no hatchery production. The condition of SRKW reputedly improves from their return to the waters of the Salish Sea in the early summer to their relative dispersal in the fall; these data indicate that this foraging period is primarily supported by wild salmon populations.

**SPECIFIC LINE EDITS**

519 – data on NRKW age of prey from Ford and Ellis 2006 is from prey remains collected in 2003-2005. To better support the observation that NRKW consume older/larger Chinook, authors may consider application of a correction factor for the 15 year lag between the NRKW and SRKW data sets presented Fig 8a and B.

555 – suggest rewording for clarity – confusing statement.

624 – bioaccumulation of PBDEs provide insight into past foraging behaviours (Krahn et al, 2007) and these data represent foraging events that have occurred prior to 2007 (and with bioaccumulation and selective mobilization of lipids and thus contaminants, they may represent foraging events from decades in the past). Suggest modifying to indicate these data support the historic foraging conditions.

Figure 8b – y axis label should read “Proportion”
